# Stigma and its associated factors among patients with COVID-19 in Dhaka City: evidence from a cross-sectional investigation

**DOI:** 10.7717/peerj.14092

**Published:** 2022-10-06

**Authors:** Md. Golam Kibria, Taslima Islam, Md. Tajul Islam, Russell Kabir, Shakil Ahmed, Papia Sultana

**Affiliations:** 1 Department of Research, Centre for Development Action, Dhaka, Bangladesh; 2 Department of Public Health, North South University, Dhaka, Bangladesh; 3 School of Allied Health, Anglia Ruskin University, Chelmsford, Essex, UK; 4 Maternal and Child Health Division, International Centre for Diarrhoeal Disease Research, Bangladesh, Dhaka, Bangladesh; 5 Department of Statistics, University of Rajshahi, Rajshahi, Bangladesh

**Keywords:** COVID-19, Stigma, Patients, Cross-sectional, Dhaka City

## Abstract

**Background:**

Coronavirus disease 2019 (COVID-19) has become a significant disease pandemic. Dhaka City alone has contributed about one-third to the total COVID-19 cases in Bangladesh. Globally, patients with infectious diseases, including COVID-19, experience stigma. There was no quantitative estimate of stigma experienced by patients with COVID-19 in the country. Therefore, this study aimed to assess the prevalence of stigma and its associated factors among patients with COVID-19 in Dhaka.

**Methods:**

A cross-sectional study was conducted among 384 respondents aged 18 years or older who had been hospitalized or had stayed at home and were tested negative 15 days to 6 months before the day of data collection. Data collection was done through in-person and telephone interviews using a semi-structured survey questionnaire. A 15-item COVID-19-related stigma scale questionnaire was used to assess stigma. Binary logistic regression analysis was performed to identify the predictors of stigma.

**Results:**

More than half (53.1%) of the respondents experienced stigma when they were COVID-19 positive. Females were at a 3.24 times higher risk of experiencing stigma than their male counterparts. Respondents from the 60+ age group and 40–59 age group were 63.0% and 48.0% less likely to experience stigma than those from the 18–39 age group. Non-hospitalised patients had 1.67 times higher odds of facing stigma than those hospitalised.

**Conclusions:**

This study reported a high prevalence of stigma among the patients with COVID-19 in Dhaka City. The current evidence base of stigma experience among patients with COVID-19 offers a solid foundation for creating effective strategies and policies and designing appropriate interventions to counter stigma, which will improve the psychological well-being of patients with COVID-19 in Bangladesh.

## Introduction

Coronavirus disease 2019 (COVID-19) has become a significant disease pandemic. This infectious disease is caused by severe acute respiratory syndrome coronavirus 2 (SARS-CoV-2) (Gorbalenya et al., 2020). COVID-19 which originated in Wuhan City of China in early December 2019 has taken the lives of more than 6 million people globally (WHO, 2022). As of 27 March 2022, Bangladesh has reported about 2 million COVID-19 cases and more than 29,000 deaths, and Dhaka City alone has contributed about one-third to the country’s total COVID-19 cases (WHO, 2022). Every year thousands of people migrate to the city from different parts of the country to search for better livelihood opportunities. Hence, Dhaka has become the country’s most populated city and the 9^th^ largest megacity in the world, with a population exceeding 19 million (United Nations, 2018).

Historically, there is a link between stigma and infectious diseases. During the Middle Ages, the plague was considered a divine punishment for people’s sinful acts (Agresta, 2020). In the 19^th^ century, Mary Mallon was forced into quarantine for a total of 26 years for spreading typhoid, and was later commonly known as Typhoid Mary, synonymous with the spread of disease (Marineli et al., 2013). People living with HIV/AIDS are often believed to deserve their HIV-positive status as a consequence of having done something ‘wrong’ (UNAIDS, 2005). A study in Hong Kong explored that SARS victims were stigmatized by their family members, friends and colleagues after their recovery (Siu, 2008). According to a study by Kelly et al. (2019), survivors of the 2013–2016 West Africa Ebola outbreak experienced social ostracization and unemployment. Similarly, the current COVID-19 pandemic has provoked stigma, discrimination and negligence toward patients with COVID-19 (Miah et al., 2022). A recent meta-analysis reported that 38.0% of patients with COVID-19 experienced stigma worldwide (Yuan et al., 2022).

Stigma is a neglectful attitude that an individual receives from others for a particular characteristic thought to be wrong or unusual, over which they have no control. In 1963, Erving Goffman described stigma as “an attribute which is deeply discrediting” that reduces a person “from a whole and usual person to a tainted, discounted one” (Goffman, 1969). The scientific community has identified several forms of stigma, including internalised stigma, disclosure concerns, enacted stigma, and perceived stigma (Earnshaw & Chaudoir, 2009; Turan et al., 2017; Sangma et al., 2022). Enacted stigma occurs when people experience prejudice, discrimination, and exclusion due to their stigmatizing condition. In contrast, internalised stigma refers to the feeling of shame and guilt resulting from the stigmatizing state (Scambler, 2004). Perceived public stigma is defined as people’s beliefs about the attitudes of others towards a particular stigmatizing attribute (Subu et al., 2021). Disclosure concerns refer to worrying about the negative consequences of disclosing the stigmatizing condition (Anakwa, Teye-Kwadjo & Kretchy, 2021). A series of factors such as misinformation, misconceptions, rumours, contagious nature of the virus, fear of infection, avoidance, bullying, threat, quarantine and lack of trust in the health system contribute to the development of stigma among patients and healthy people during infectious disease outbreaks (Miah et al., 2022; WHO, 2020; Mahmud & Islam, 2021). Stigma exerts adverse effects not only on the mental health of victims but also on their physical health and social and economic status. Patients with COVID-19 were rejected by their family members, relatives, neighbours, and colleagues during the early stages of the pandemic (The Business Standard, 2020; Deccan Chronicle, 2022; Amir, 2021). In Bangladesh, incidents like frontline doctors and nurses being pressured to vacate their rented apartments were reported all over the country (The Financial Express, 2020). A study in Saudi Arabia showed that stigma led to adverse psychological outcomes, including depression and anxiety among COVID-19 patients (Alkathiri et al., 2022). Stigma poses a significant obstacle to the control and prevention of COVID-19. Evidence shows that patients with COVID-19 symptoms refused diagnostic tests and medical treatment to avoid being stigmatized and marginalized in society (Siam et al., 2021; Villa et al., 2020). This type of health-seeking behaviour not only increases patients’ health risk but also facilitates the spread of the virus.

To the best of our knowledge, there was no quantitative estimate of stigma experienced by patients with COVID-19 in Bangladesh. About one-third of the country’s COVID patients live in Dhaka city. Taking into account the evidence of the psychological distress in patients with infectious diseases, we hypothesized that a significant portion of patients with COVID-19 in Dhaka city would experience stigma. Assessing the prevalence of stigma and its association with other factors is essential because this would help to understand the severity of the problem and design appropriate stigma reduction interventions for the betterment of patients with COVID-19. Therefore, this study aimed to assess the prevalence of stigma and identify its associated factors among patients with COVID-19 in Dhaka City. Throughout this article, we used the term ‘stigma’ to denote overall stigma.

## Materials and Methods

### Study design and participants

This cross-sectional study was conducted among COVID-19 survivors living in Dhaka city in Bangladesh from 19 January to 30 June 2021. Study participants included patients with COVID-19 aged 18 years or older who had been hospitalised or had stayed at home and were tested negative 15 days to 6 months before data collection. Patients with speech and/or hearing impairments were excluded so that we might not have difficulty gathering information from those patients.

### Procedures

For this study, we recruited two types of patients with COVID-19–hospitalised and non-hospitalised, who had recovered over 15 days to 6 months, from 1^st^ November 2020 to 15^th^ April 2021. We chose hospitalised and non-hospitalised patients to see differences in the prevalence rate of stigma between the groups. Because of administrative difficulties and confidentiality issues, we could collect the medical records of two hospitals in Dhaka City. One was a government COVID-19 dedicated hospital, and the other was a private hospital with a COVID-19 unit. We collected the medical records of 500 inpatients from these two hospitals. We selected 192 patients from these 500 hospitalised patients following the simple random sampling technique and the other 192 non-hospitalised patients using the snowball sampling technique. We communicated with all the selected patients over the phone and fixed the interview time at least 3 days before the interview. As some respondents were not interested in joining the in-person interview for fear of contracting COVID-19, we also employed the telephone interview mode. We recruited data collectors with medical science and social science backgrounds and gave them 2-day training before data collection. However, data collection was done through in-person and telephone interviews (282 *vs*. 102) using a semi-structured survey questionnaire. Each interview lasted for approximately 20 minutes. Almost all the in-person interviews were conducted in the respondents’ houses, and a few took place in their offices. Safety measures such as physical distancing and face mask use were strictly maintained during the time of in-person interviews. However, the respondents were not given any financial benefits for participating in the study.

### Measures

#### Basic information

The basic information of the study respondents was collected on sex, age, marital status, education, occupation, religion, monthly family income and hospitalisation status.

### COVID-19-related stigma scale

In this study, stigma was measured using a 15-item stigma scale questionnaire. Dar et al. (2020) adapted the questionnaire from the Ebola-related stigma scale (Overholt et al., 2018) for use among patients with COVID-19 in Kashmir, India. The Ebola-related stigma scale was derived from Berger’s HIV stigma scale (Wright et al., 2007), a validated measure of stigma for people living with HIV. This stigma scale includes four subscales-enacted stigma (three items), disclosure concerns (two items), internalised stigma (three items) and perceived external stigma (seven items). Each item is rated on a 4-point Likert scale (0 = strongly disagree, 1 = disagree, 2 = agree, 3 = strongly agree), with total scores ranging from 0 to 45. Higher scores indicate greater experiences of stigma. The respondents scoring greater than or equal to the median score of 23 were classified as having experienced stigma. In contrast, those scoring less than the median score of 23 were classified as not having experienced stigma (Sima, Belachew & Abebe, 2019). The final COVID-19-related stigma questionnaire that we used in our study is shown in Table 1. The English version questionnaire was translated, back-translated into and from Bengali and pretested among 20 COVID-19 survivors for our research. The Bengali version COVID-19-related stigma scale had good reliability (Cronbach’s alpha = 0.857).

**Table 1:** COVID-19-related stigma items.

Stigma subscales and items
**Enacted stigma**
1. I have been hurt by how people reacted to learning I had coronavirus disease.
2. I have stopped socializing with some people because of their reactions of my having had coronavirus disease.
3. I have lost friends because I had coronavirus disease.
**Disclosure concerns**
4. I am very careful who I tell that I had coronavirus disease.
5. I worry that people who know I have had coronavirus disease will tell others.
**Internalised stigma**
6. I feel that I am not as good as a person as others because I had coronavirus disease.
7. Having had a COVID-19 infection makes me feel that I am a bad person.
8. I feel guilty because I am COVID-19 positive.
**Perceived external stigma**
9. Most people think that a person who has had coronavirus disease is disgusting.
10. Most people are afraid of a person who has had coronavirus disease.
11. Most people who have had coronavirus disease are rejected when others find out.
12. People I know would treat someone who has had coronavirus disease as an outcast.
13. People I know would be uncomfortable around someone who has had coronavirus disease.
14. People I know would reject someone who has had coronavirus disease.
15. People I know would not want someone who has had coronavirus disease around their children.

**Note:**

Stigma category headings were not used in the administered survey questionnaire.

### Sample size calculation

Probability sampling was used to calculate the sample size of this study. The sample size was calculated using the following formula:

n = $\displaystyle{{{z^2}pq} \over {{d^2}}}$; n = $\displaystyle{{{{1.96}^2} \times (1 - 0.50)} \over {{{0.05}^2}}}$ = 384.16 ≈ 384

Here,

n = Desired sample size

z = Standard normal deviate = 1.96 at 95% confidence interval

p = Prevalence estimate = 50% = 0.50 as there was no reported prevalence of stigma among COVID-19 patients in Dhaka city at the time when this study was designed.

q = 1 − p = 1 − 0.50 = 0.50

d = Precision of the prevalence estimate = 5% = 0.05

The calculated sample size of this study was 384.

### Statistical analysis

Data analysis was performed using SPSS statistical software version 25. For descriptive statistics, frequency and percentage were used to describe the basic characteristics of the respondents. Median and interquartile range (IQR) were calculated for the stigma scale and subscales. We checked for normality of the stigma scale and subscale scores using the Shapiro-Wilk test, and the data did not meet normality (*p* < 0.05). Therefore, we used the nonparametric Mann-Whitney U test and Kruskal-Wallis test to compare stigma scale and subscale score differences between groups. Binary logistic regression analyses were done with variables associated with the total stigma score at *p* < 0.20 in the univariate analysis. These variables included sex, age, education, occupation, monthly family income, and hospitalisation status. The backward stepwise logistic regression method was used to identify the predictors of stigma. The associations between predictors and outcome variables were presented as adjusted odds ratios (ORs) and 95% CIs. The regression model met the requirements of both the omnibus test (*p*-value < 0.05) and the Hosmer-Lemeshow test for goodness of fit (*p*-value *>* 0.05). All statistics were tested using a two-sided test, and a *p*-value of <0.05 was considered statistically significant.

### Ethical considerations

Formal ethical approval was obtained from the national research ethics body, Bangladesh Medical Research Council (BMRC) (Ref: BMRC/HPNSP-Research Grant l2020-2021 I 52(1-47). We did not disclose the identities of the two hospitals because the authorities assumed that this could put these hospitals at risk of stigmatization. Written informed consent was taken from those who joined the in-person interview, and verbal informed consent was sought from the respondents participating in the phone interview. Confidentiality of the information given by the respondents was strictly maintained. The informed consent form clearly explained the aims and procedures of the study, risks and benefits associated with participation, their right to voluntary participation and their right to withdraw from the study, and anonymity and confidentiality of their data.

## Results

### Basic characteristics of the respondents

The distribution of respondents by basic characteristics is shown in Table 2. Most respondents (57.8%) were males, whereas 42.2% were females. 43.0% of the respondents belonged to the 18–39 age group, 35.7% belonged to the 40–59 age group, and 21.4% belonged to the 60+ age group. The vast majority of the respondents (80.2%) were married, 18.2% were unmarried and only 1.6% were divorced/widowed. Overall, the educational status of the respondents was very high. More than three-fourths of the respondents (78.9%) completed Bachelor’s degree and more education, 12.0% completed higher secondary education, 4.9% completed secondary education, and 4.2% had no education or completed primary education. Most respondents were service holders (41.1%), followed by housewives (22.4%). By religion, 92.7% of the respondents were Muslims, 6.5% were Hindus, and a few were Buddhists. About half of the respondents (50.3%) had a monthly family income of BDT <50,000, 33.1% had a monthly family income BDT 50,000 to 99,000, and 16.7% had a monthly income of BDT 100,000 and above. By hospitalisation status, 50.0% of the respondents were hospitalised and the remaining 50.0% were non-hospitalised during the time they were COVID-19 positive.

**Table 2:** Distribution of the respondents by basic characteristics.

Variable	Category	Frequency	Percent
Sex	Male	222	57.8
	Female	162	42.2
Age	18–39 years	165	43.0
	40–59 years	137	35.7
	60+ years	82	21.4
Marital status	Unmarried	70	18.2
	Married	308	80.2
	Divorced/widowed	6	1.6
Education	None/Primary education	16	4.2
	Secondary education	19	4.9
	Higher secondary education	46	12.0
	Bachelor’s and above	303	78.9
Occupation	Service holder	158	41.1
	Housewife	86	22.4
	Retired person	45	11.7
	Businessmen	32	8.3
	Health professional^a^	32	8.3
	Student	31	8.1
Religion	Muslim	356	92.7
	Hindu	25	6.5
	Buddhist	3	0.8
Monthly family income	<50,000 BDT	193	50.3
	50,000–99,000 BDT	127	33.1
	≥100,000 BDT	64	16.7
Hospitalisation status	Yes	192	50.0
	No	192	50.0

**Note:**

a Health professionals comprise medical doctors, nurses, physiotherapists and other hospital staff.

### Comparison of stigma levels between different groups

Table 3 shows the median scores of stigma scale and subscales among the respondents by basic characteristics. The median (IQR) scores of stigma, enacted stigma, disclosure concerns, internalised stigma and externalised stigma were 23 (21–26), 5.5 (4–6), 3 (2–4), 4 (3–5) and 12 (10–13), respectively. More females than their male counterparts experienced stigma (median = 24, IQR = 22–28), enacted stigma (median = 6, IQR = 5–6), disclosure concerns (median = 3, IQR = 2–4), internalised stigma (median = 4, IQR = 3–5) and externalised stigma (median = 12, IQR = 11–14). Stigma, enacted stigma and disclosure concerns were most prevalent among the respondents from the 18–39 age group (median = 25, IQR = 22–27; median = 6, IQR = 5–6 and median = 3, IQR = 2–4, respectively), whereas internalised stigma was reported highest by the respondents from both the 18–39 age group (median = 4, IQR = 3–5) and the 40–59 age group (median = 4, IQR = 3–5), and externalised stigma was most prevalent among the respondents from the 18–39 age group (median = 12, IQR = 11–13), the 40–59 age group (median = 12, IQR = 10–13) and the 60+ age group (median = 12, IQR = 10–13). By the education category, stigma was highest among those who completed higher secondary education (median = 23, IQR = 21–25.2) and Bachelor’s degree and above (median = 23, IQR = 21–26). In contrast, enacted stigma was experienced most by those who completed Bachelor’s degree and above (median = 6, IQR = 5–6). By occupation, stigma and externalised stigma were reported highest by health professionals (median = 25, IQR = 22–28.7 and median = 12.5, IQR = 11–14, respectively). On the other hand, enacted stigma was faced most by service holders (median = 6, IQR = 4–6), housewives (median = 6, IQR = 5–6.2) and health professionals (median = 6, IQR = 5–6). Stigma was experienced most by the respondents whose monthly family income was BDT 100,000 and above (median = 24.5, IQR = 21–27), and enacted stigma was reported highest by the respondents whose monthly family income was BDT 50,000–99,000 (median = 6, IQR = 5–6) and BDT 100,000 and above (median = 6, IQR = 5–6). By hospitalisation status, more non-hospitalised patients compared to their hospitalised counterparts were found to experience stigma (median = 24, IQR = 22–27), enacted stigma (median = 6, IQR = 5–6), disclosure concerns (median = 3, IQR = 2–4) and externalised stigma (median = 12, IQR = 11–13).

**Table 3:** Median scores of stigma scale and subscales among the respondents by basic characteristics.

Variable	Category	Stigma	Enacted stigma	Disclosure concerns	Internalised stigma	Externalised stigma
		**Median (IQR)**	**Median (IQR)**	**Median (IQR)**	**Median (IQR)**	**Median (IQR)**
Overall		23 (21–26**)**	5.5 (4–6)	3 (2–4)	4 (3–5)	12 (10–13)
Sex	Male	22 (20.7–25)	5 (4–6)	2 (2–3)	3 (3–5)	11 (9–13)
	Female	24 (22–28)	6 (5–6)	3 (2–4)	4 (3–5)	12 (11–14)
	*p*-value**^b^**	<0.001	0.001	0.004	0.023	<0.001
Age	18–39 years	25 (22–27)	6 (5–6)	3 (2–4)	4 (3–5)	12 (11–13)
	40–59 years	22 (21–25)	5 (4–6)	2 (2–4)	4 (3–5)	12 (10–13)
	60+ years	22 (20–24)	5 (4–6)	2 (2–4)	3 (3–4)	12 (10–13)
	*p*-value^c^	<0.001	0.015	0.073	0.010	0.010
Marital status	Unmarried	24.5 (21–27)	6 (5–6)	3 (2–4)	4 (3–5)	12 (11–13)
	Married	23 (21–26)	5 (4–6)	3 (2–4)	4 (3–5)	12 (10–13)
	Divorced/widowed	22 (19.2–32)	6 (6–7)	2.5 (1.5–4.2)	4.5 (1.5–6)	11 (8.5–15)
	*p*-value^c^	0.269	0.078	0.687	0.431	0.534
Education	None/Primary education	20.5 (20–26.2)	4 (3–5.7)	2 (2–3.7)	3.5 (2–4.7)	12 (10.2–14)
	Secondary education	22 (21–22)	5 (3–6)	2 (2–3)	3 (3–4)	11 (9–12)
	Higher secondary education	23 (21–25.2)	5 (4–6)	3 (2–3.2)	4 (3–5)	12 (9–13)
	Bachelor’s and above	23 (21–26)	6 (5–6)	3 (2–4)	4 (3–5)	12 (10–13)
	*p*-value^c^	0.026	0.009	0.411	0.319	0.112
Occupation	Service holder	23 (21–26)	6 (4–6)	3 (2–4)	4 (3–5)	12 (10–13)
	Housewife	24 (21.7–29)	6 (5–6.2)	3 (2–4)	4 (3–5)	12 (11–14)
	Retired person	22 (20–23)	5 (4–6)	2 (2–3)	3 (3–4.5)	11 (7–12)
	Businessmen	22 (21–24.7)	5 (4–6)	2.5 (2–3)	3 (3–5)	12 (10.2–13)
	Health professional	25 (22–28.7)	6 (5–6)	3 (2–4)	4.5 (3–6)	12.5 (11–14)
	Student	24.0 (21–25)	5 (5–6)	3 (2–4)	3 (3–5)	12 (11–13)
	*p*-value^c^	<0.001	0.029	0.482	0.056	0.004
Religion	Muslim	23 (21–26)	5 (4–6)	3 (2–4)	4 (3–5)	12 (10–13)
	Hindu	22 (21–26)	6 (4.5–6)	3 (2–3)	4 (3–5)	11 (10–13)
	Buddhist	23 (-----)	6 (-----)	3 (-----)	4 (-----)	10 (-----)
	*p*-value^c^	0.974	0.916	0.477	0.812	0.600
Monthly family income	<50,000 BDT	22 (21–25.5)	5 (4–6)	3 (2–4)	4 (3–5)	12 (10–13)
	50,000–99,000 BDT	24 (22–27)	6 (5–6)	3 (2–4)	4 (3–5)	12 (10–13)
	≥100,000 BDT	24.5 (21–27)	6 (5–6)	2 (2–3.7)	4 (3–5)	12 (11–13)
	*p*-value^c^	0.033	0.028	0.454	0.395	0.107
Hospitalisation status	Yes	22 (20–24.7)	5 (4–6)	2 (2–3)	4 (3–5)	11 (9.2–13)
	No	24 (22–27)	6 (5–6)	3 (2–4)	4 (3–5)	12 (11–13)
	*p*-value^b^	<0.001	<0.001	0.022	0.072	<0.001

**Notes:**

b Mann-Whitney U test.

c Kruskal-Wallis test.

IQR, Interquartile range; (-----), IQRs were not obtained due to an inadequate number of observations.

### Individual responses to stigma-related items

**Figure 1:**
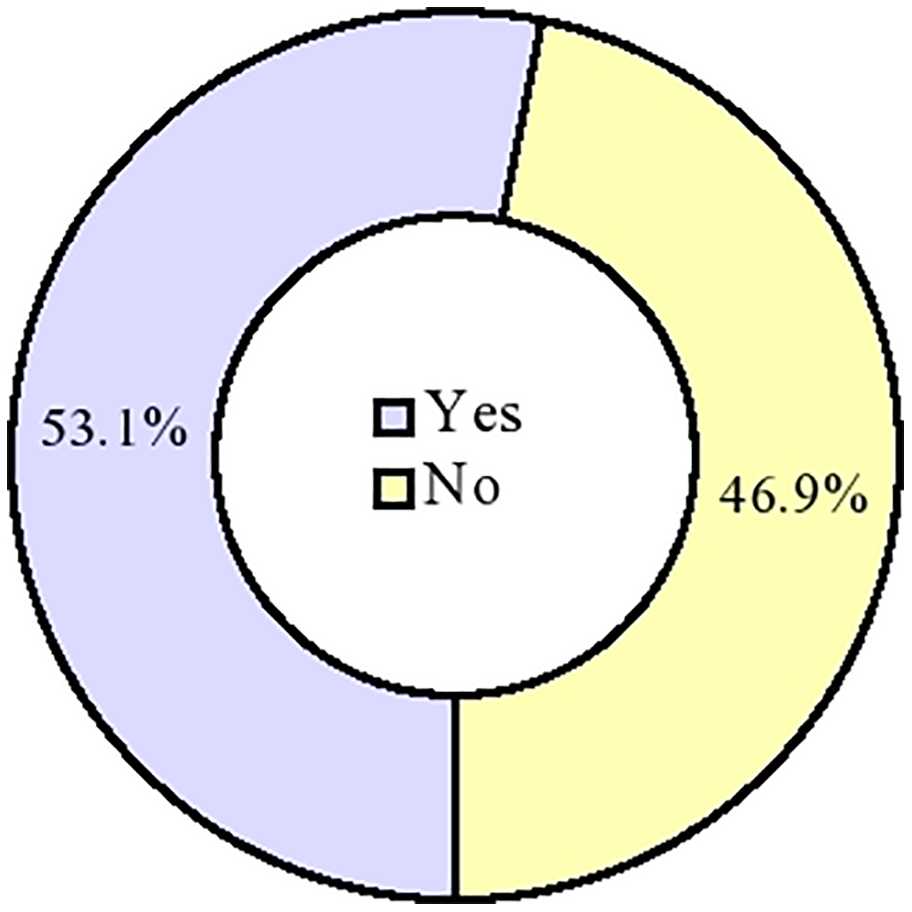
Experience of COVID-19-related stigma among the respondents. Experience of stigma (*n* = 384).

Individual responses of the respondents to stigma-related items are shown in Fig. 1. 84.9% and 72.9% of the respondents agreed/strongly agreed with two out of three items of the enacted stigma subscale (‘hurt by how people reacted to my coronavirus disease’ and ‘I have stopped socialising with some people’, respectively). 86.2%, 79.7%, 74.5%, 68.0% and 62.8% of the respondents agreed/strongly agreed with five out of seven items of the externalised stigma subscale. On the other hand, the least proportion of respondents agreed/strongly agreed with two out of three items of the internalised stigma subscale– ‘a person with coronavirus disease is disgusting’ (18.8%) and ‘I feel that I am not as good a person as others’ (20.6%).

### Experience of stigma among the respondents

**Figure 2:**
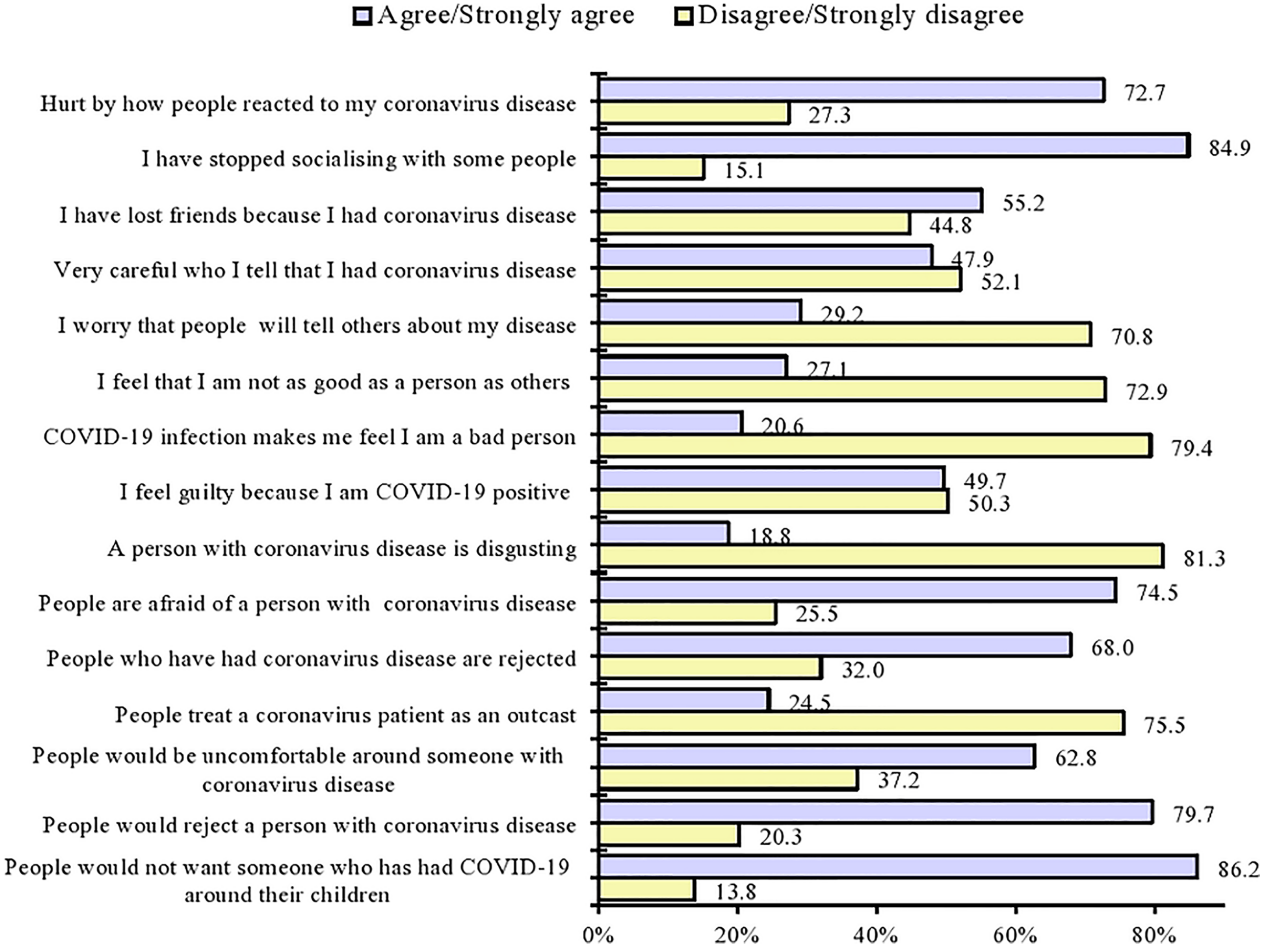
Individual responses to stigma-related items by the respondents.

As shown in Fig. 2, 53.1% (*n* = 204) of the respondents experienced some level of stigma when they were COVID-19 positive, whereas 46.9% (*n* = 180) of the respondents did not experience stigma when they were COVID-19 positive.

### Predictors of stigma

The binary logistic regression analysis results presented in Table 4 describe that the females had a 3.24 times higher chance of experiencing stigma than their male counterparts (95% CI [2.039–5.163]; *p* < 0.001) when they were COVID-19 positive. The respondents from the 60+ age group and the 40–59 age group were 63.0% (95% CI [0.199–0.696]; *p* = 0.015) and 48.0% (95% CI [0.311–0.880]; *p* = 0.002) less likely to experience stigma than those from the 18–39 age group. By hospitalisation status, the non-hospitalised patients had 1.67 times higher odds of facing stigma than the hospitalised patients (95% CI [1.033–2.727]; *p*-value = 0.037).

**Table 4:** Association between stigma and basic characteristics of the respondents.

Variable	Category	B	Wald	Adjusted OR	95% CI	*p*-value
Sex	Male			Reference		
	Female	1.177	24.672	3.24	[2.039–5.163]	<0.001***
Age	18–39 years			Reference		
	40–59 years	−0.648	5.962	0.52	[0.311–0.880]	0.015*
	60+ years	−0.989	9.574	0.37	[0.199–0.696]	0.002**
Hospitalisation status	Yes			Reference		
	No	0.518	4.369	1.67	[1.033–2.727]	0.037*

**Notes:**

* *p* < 0.05.

** *p* < 0.01.

*** *p* < 0.001.

OR, Odds Ratio; CI, Confidence Interval.

## Discussion

This study explored the experience of stigma among the patients with COVID-19 in Dhaka City and identified the factors associated with stigma. The present study revealed that 53.1% of the respondents experienced stigma when they were COVID-19 positive. Available evidence shows that stigma among patients with COVID-19 ranged from 43.3% to 70.2% in different countries (Sugiyama et al., 2022; Haddad et al., 2021; Wahyuhadi et al., 2022). A study conducted among 502 quarantined and isolated patients with COVID-19 in Bahrain found that 53.4% of the respondents experienced stigma (Jassim et al., 2021). A study of 509 Egyptian healthcare workers revealed that 57.5% of the respondents reported self-stigma (Mostafa, Sabry & Mostafa, 2020). Some of the variations in the prevalence of stigma could be attributed to study designs, data collection tools, study populations, study time and cultures that differed from one study to another. However, the findings suggest that stigma is a common global concern among patients with COVID-19, and this group urgently needs mental health services and support.

In the present study, sex was significantly associated with COVID-19-related stigma. The study’s findings showed that females were more likely to experience stigma than their male counterparts. This finding is consistent with previous studies carried out among patients with infectious diseases, including COVID-19, showing a higher prevalence of stigma among females than males (Zandifar et al., 2020; James et al., 2019; Jarolimova et al., 2021). Several factors could be attributed to the higher prevalence of stigma in females. First, females in our culture are less likely than males to protest discrimination, stigma and abuse committed against them, which gives other people a scope to show discriminatory and stigmatizing behaviours towards them. Second, females in the country have less access to information than males (Asian Development Bank, 2017). This limited access heightens their vulnerability to myths, rumours and misinformation regarding COVID-19, which may have provoked feelings of stigmatization in them. Furthermore, the higher prevalence of stigma in females could be partly due to fluctuations in the levels of ovarian hormones in them during exposure to emotional stimuli that they experience (Soni, Curran & Kamboj, 2013). Therefore, stigma reduction interventions should be designed for patients with COVID-19 with particular attention to the female sex.

According to the findings of the present study, middle-aged (40–59 years) and older adults (60 years and above) were less likely to experience COVID-19-related stigma than their young counterparts (18–39 years). This finding is supported by a study conducted in China, which showed that the patients with COVID-19 aged 50 years or older experienced lower levels of stigma than those aged below 50 years (Lin et al., 2021). Another study in Liberia among Ebola virus disease survivors found that older adults were at a lower risk of stigma than those aged 20–49 years (Kelly et al., 2019). Similar findings have also been reported in other studies investigating the level of stigma among patients with mental health illnesses (Nugent et al., 2021; Mackenzie et al., 2019). One possible explanation for the lower prevalence of stigma among older adults is that they are more skilled at emotion regulation in stressful situations than young adults (Knight et al., 2007; Urry & Gross, 2010; Abuhammad, Alzoubi & Khabour, 2021). Therefore, they may have developed lower levels of stigma when they were COVID-19 positive. The findings suggest the need for age-specific stigma reduction interventions for patients with COVID-19, focusing on young adults.

Available literature suggests that more hospitalised patients than non-hospitalised patients experienced disease-related stigma (Tesfaw, Kibru & Ayano, 2020; Ju et al., 2022). But the present study did not agree with this finding showing that non-hospitalised patients with COVID-19 had a higher risk of stigma than those with COVID-19. This higher prevalence could be explained by the fact that non-hospitalised patients do not receive appropriate healthcare and counselling compared to hospitalised patients. Evidence suggests that psychosocial counselling can help patients optimize their mental health (National Institute of Mental Health and Neurosciences, 2020). Due to lack of counselling, the non-hospitalised patients may have developed feelings of fear and stigma. Therefore, destigmatizing counselling should be integrated into the country’s national telehealth services, targeting the psychological wellbeing of non-hospitalised COVID-19 patients.

## Limitations

This study has some limitations. First, the study followed a cross-sectional design that cannot establish a temporal relationship between outcome and independent variables because both variables were observed simultaneously. Second, the data were collected from the patients with COVID-19 several weeks after their recovery, which increased the risk of recall bias. Third, we randomly selected the hospitalized patients and the non-hospitalised patients using snowball sampling. The mixture of probability and non-probability sampling limits the generalization of the findings. Fourth, we did not assess the respondents’ other psychological outcomes, such as anxiety, depression, and post-traumatic stress disorder (PTSD). These factors may have affected the findings. Finally, this study used phone interviews to collect data from many respondents. The absence of body language in the phone interview might cause poor communication between the interviewer and the respondent, affecting the findings of this study.

## Conclusions

This study reported a high prevalence of stigma among the patients with COVID-19 in Dhaka City. The study revealed that female sex, middle and old age and hospitalisation were significantly associated with COVID-19-related stigma. The findings highlight the necessity of providing psychological services for female, middle-aged and older non-hospitalised patients with COVID-19. However, the current evidence base of stigma experience among patients with COVID-19 offers a solid foundation for creating effective strategies and policies and designing appropriate interventions to counter stigma, which will improve the psychological well-being of patients with COVID-19 in Bangladesh.

## Supplemental Information

10.7717/peerj.14092/supp-1 Supplemental Information 1 Questionnaire of study.

10.7717/peerj.14092/supp-2 Supplemental Information 2 Raw data.

10.7717/peerj.14092/supp-3 Supplemental Information 3 Codebook for raw data.

## Additional Information and Declarations

### Data Availability

The following information was supplied regarding data availability: The raw data are available in the Supplemental File.
