# Supplementary material for: Stigma and its associated factors among patients with COVID-19 in Dhaka City: evidence from a cross-sectional investigation
Source: PeerJ. 2022 Oct 6;10:e14092. doi: 10.7717/peerj.14092 (PMC9548314; doi:10.7717/peerj.14092)
Supplement: Supplemental Information 1 [file peerj-10-14092-s001.docx]

**Questionnaire of the study**

| **Basic Information** | | | | | | |
| --- | --- | --- | --- | --- | --- | --- |
| **Ques No.** | **Questions** | **Options and code** | | | | |
| 1.1 | Sex | 1. Male 2*.* Female | | | | |
| 1.2 | What is your age | _______Years | | | | |
| 1.3 | What is your marital status? | 1.Unmarried  2.Married  3.Divorced/widowed  4. Living separately | | | | |
| 1.4 | What is your highest level of education? | 1.None/Primary education  2.Secondary education  3.Higher secondary education  4.Bachelor’s degree and above | | | | |
| 1.5 | What is your occupation? | 1. Service holder 2. Housewife 3. Retired person 4. Businessmen 5. Health professional 6. Student   7. Other_____________ | | | | |
| 1.6 | How much is your monthly family income? | ________ BDT | | | | |
| 1.7 | What is your religion? | 1. Muslim 2. Hindu 3. Christian   4.Buddhist | | | | |
| 1.8 | Were you admitted to the hospital when you were COVID-19 positive | 1=Yes  2=No | | | | |
| **COVID-19-Related Stigma** | | | | | | |
|  | What type of stigma did you experience when you were COVID-19 positive? | | | | | |
|  | Please circle only one response. | | Strongly disagree | Disagree | Agree | Strongly agree |
| 2.1 | I have been hurt by how people reacted to learning I had coronavirus disease. | | 0 | 1 | 2 | 3 |
| 2.2 | I have stopped socializing with some people because of their reactions of my having had coronavirus disease. | | 0 | 1 | 2 | 3 |
| 2.3 | I have lost friends because I had coronavirus disease. | | 0 | 1 | 2 | 3 |
| 2.4 | I am very careful who I tell that I had coronavirus disease. | | 0 | 1 | 2 | 3 |
| 2.5 | I worry that people who know I have had coronavirus disease will tell others. | | 0 | 1 | 2 | 3 |
| 2.6 | I feel that I am not as good as a person as others because I had coronavirus disease. | | 0 | 1 | 2 | 3 |
| 2.7 | Having had COVID-19 infection makes me feel that I am a bad person. | | 0 | 1 | 2 | 3 |
| 2.8 | I feel guilty because I am COVID-19 positive. | | 0 | 1 | 2 | 3 |
| 2.9 | Most people think that a person who has had coronavirus disease is disgusting. | | 0 | 1 | 2 | 3 |
| 2.10 | Most people are afraid of a person who has had coronavirus disease. | | 0 | 1 | 2 | 3 |
| 2.11 | Most people who have had coronavirus disease are rejected when others find out. | | 0 | 1 | 2 | 3 |
| 2.12 | People I know would treat someone who has had coronavirus disease as an outcast. | | 0 | 1 | 2 | 3 |
| 2.13 | People I know would be uncomfortable around someone who has had coronavirus disease. | | 0 | 1 | 2 | 3 |
| 2.14 | People I know would reject someone who has had coronavirus disease. | | 0 | 1 | 2 | 3 |
| 2.15 | People I know would not want someone who has had coronavirus disease around their children. | | 0 | 1 | 2 | 3 |
