# Supplementary material for: Stigma and its associated factors among patients with COVID-19 in Dhaka City: evidence from a cross-sectional investigation
Source: PeerJ. 2022 Oct 6;10:e14092. doi: 10.7717/peerj.14092 (PMC9548314; doi:10.7717/peerj.14092)
Supplement: Supplemental Information 3 [file peerj-10-14092-s003.docx]

# Supplementary Table 1. Values of categories variables.

| **Variables** | **Values** |
| --- | --- |
| **Basic Information** |  |
| Sex | 1=Male  2=Female |
| Age | 1=18-39 years  2=40-59 years  3=60-70 years |
| Marital status | 1=Unmarried  2=Married  3=Divorced/widowed |
| Education | 1=None/Primary education  2=Secondary education  3=Higher secondary education  4=Bachelor’s degree and above |
| Occupation | 1=Service holder  2=Housewife  3=Retired person  4=Businessmen  5=Health professional  6=Student  7=Other |
| Religion | 1=Muslim  2=Hindu  3=Christian  4=Buddhist |
| Monthly family income | 1=<50,000 BDT  2=50,000-99,000 BDT  3=≤100,000 BDT |
| Hospitalization status | 1=Yes  2=No |
| I have been hurt by how people reacted to learning I had coronavirus disease. | 0=Strongly disagree  1=Disagree  2=Agree  3=Strongly agree |
| I have stopped socializing with some people because of their reactions of my having had coronavirus disease. | 0=Strongly disagree  1=Disagree  2=Agree  3=Strongly agree |
| I have lost friends because I had coronavirus disease. | 0=Strongly disagree  1=Disagree  2=Agree  3=Strongly agree |
| I am very careful who I tell that I had coronavirus disease. | 0=Strongly disagree  1=Disagree  2=Agree  3=Strongly agree |
| I worry that people who know I have had coronavirus disease will tell others. | 0=Strongly disagree  1=Disagree  2=Agree  3=Strongly agree |
| I feel that I am not as good as a person as others because I had coronavirus disease. | 0=Strongly disagree  1=Disagree  2=Agree  3=Strongly agree |
| Having had COVID-19 infection makes me feel that I am a bad person. | 0=Strongly disagree  1=Disagree  2=Agree  3=Strongly agree |
| I feel guilty because I am COVID-19 positive. |  |
| Most people think that a person who has had coronavirus disease is disgusting. | 0=Strongly disagree  1=Disagree  2=Agree  3=Strongly agree |
| Most people who have had coronavirus disease are rejected when others find out. | 0=Strongly disagree  1=Disagree  2=Agree  3=Strongly agree |
| People I know would treat someone who has had coronavirus disease as an outcast. | 0=Strongly disagree  1=Disagree  2=Agree  3=Strongly agree |
| People I know would be uncomfortable around someone who has had coronavirus disease. | 0=Strongly disagree  1=Disagree  2=Agree  3=Strongly agree |
| People I know would reject someone who has had coronavirus disease. | 0=Strongly disagree  1=Disagree  2=Agree  3=Strongly agree |
| People I know would not want someone who has had coronavirus disease around their children. | 0=Strongly disagree  1=Disagree  2=Agree  3=Strongly agree |
